# Detection of *Plum pox virus* and Characterization of Oxidative Stress Responses in Stone Fruit Trees of Azerbaijan

**DOI:** 10.3390/ijms27219406

**Published:** 2026-10-22

**Authors:** Nargiz Sultanova, Elshan Musazade, Serife Topkaya

**Affiliations:** 1 Department of Biotechnology and Food Engineering, Azerbaijan State Oil and Industry University, AZ1010 Baku, Azerbaijan; 2 Key Laboratory of Soybean Molecular Design Breeding, Northeast Institute of Geography and Agroecology, Chinese Academy of Sciences, Changchun 130102, China; 3 Department of Agriculture, Tokat Gaziosmanpasa University, 60250 Tokat, Türkiye; serife.topkaya@gop.edu.tr

**Keywords:** *Plum pox virus*, *Prunus* species, oxidative stress, catalase, ascorbate peroxidase, chlorophyll degradation, sustainable agriculture, plant health, crop protection

## Abstract

Sharka disease, induced by the *Plum pox virus* (PPV; genus *Potyvirus*, family *Potyviridae*), represents one of the most serious viral threats to stone fruit production worldwide. This study assessed PPV occurrence in major fruit-growing areas in Azerbaijan and characterized the physiological and oxidative stress responses of infected *Prunus* species. Symptomatic leaves from orchards in the Quba district were screened by immunochromatographic strips, DAS-ELISA, and RT-PCR. Of 60 trees surveyed, 22 (36.7%) tested positive across all three hosts, confirmed by RT-PCR amplification of a 243 bp coat protein (CP) gene fragment in peach (*Prunus persica* L.), plum (*P. domestica* L.), and apricot (*P. armeniaca* L.). Infection was associated with pronounced oxidative stress: hydrogen peroxide (H_2_O_2_) and malondialdehyde (MDA) contents were higher in infected trees (6.80–10.60 µmol g^−1^ FW and 8.0–15.2 nmol g^−1^ FW) than in healthy trees (3.20–3.90 µmol g^−1^ FW and 4.5–8.2 nmol g^−1^ FW). Antioxidant defenses were activated, with increased ascorbate peroxidase (APX) and catalase (CAT) activities and higher ascorbic acid (AsA) and reduced glutathione (GSH) levels. Photosynthetic performance declined markedly: total chlorophyll fell by 17.3–30.8% (greatest in apricot), maximum photosystem II quantum efficiency (F_v_/F_m_) was lower in infected trees (0.408–0.561) than in healthy trees (0.716–0.728), and total soluble protein was lower in infected trees (7.88–8.45 mg g^−1^ FW) than in healthy trees (9.38–9.58 mg g^−1^ FW). Collectively, PPV infection was associated with oxidative stress and lipid peroxidation, with activation of enzymatic and non-enzymatic antioxidant defenses, and with reduced photosynthetic performance in *Prunus* species. These findings advance understanding of host responses to PPV and support sustainable monitoring and management strategies for stone fruit production, aligned with SDG 2 (Zero Hunger) and SDG 15 (Life on Land).

## 1. Introduction

Stone fruit trees of the genus *Prunus* (family *Rosaceae*, subfamily *Prunoideae*) are among the most economically and nutritionally important groups of perennial fruit crops worldwide. The genus comprises more than 230 species, traditionally divided into the subgenera *Amygdalus*, *Cerasus*, and *Prunus*, together with the desert-adapted subgenus *Emplectocladus* [1]. These species bear drupaceous fruits that are consumed fresh or processed and contribute substantially to global fruit production and agricultural economies. According to the Food and Agriculture Organization of the United Nations (FAO), global stone fruit production reached approximately 50 million tons in 2023, with peach (*Prunus persica* (L.) Batsch) and nectarine as the dominant crops, accounting for nearly half of total output. Peach alone exceeded 27 million tons harvested from more than 1.5 million hectares, underscoring its global agronomic importance [2].

Beyond their economic value, stone fruits are recognized as functional foods because of their rich biochemical composition. Peach and related *Prunus* species contain a broad spectrum of bioactive compounds, including vitamin C (ascorbic acid, AsA), carotenoids, flavonoids, phenolic acids, and other polyphenols with well-documented antioxidant, anti-carcinogenic, and anti-inflammatory properties. Carotenoids and AsA contribute substantially to reactive oxygen species (ROS) scavenging, whereas polyphenols participate in metal chelation and radical neutralization. Consequently, *Prunus* fruits are considered important dietary sources of natural antioxidants [1,3].

Plant–virus interactions constitute a major biological system in which oxidative stress plays a central role. Agricultural systems are continuously exposed to abiotic stresses such as salinity, drought, and temperature extremes, as well as biotic stresses caused by viruses, fungi, and bacteria. Among these, viral infection is one of the most important constraints on agricultural productivity. *Plum pox virus* (PPV), the causal agent of Sharka disease, is regarded as the most devastating viral pathogen of stone fruit trees. PPV belongs to the genus *Potyvirus* (family *Potyviridae*) and infects a wide range of *Prunus* species, including plum (*Prunus domestica*), apricot (*P. armeniaca*), peach (*P. persica*), almond (*P. dulcis*), and nectarine [3]. Since its first description in the early twentieth century, PPV has spread across Europe, Asia, Africa, and parts of the Americas, causing severe economic losses in stone fruit production. Infection can reduce yield by up to 80%, depending on cultivar susceptibility, viral strain, environmental conditions, and the timing of infection [4]. Infected fruits frequently become deformed, develop chlorotic rings or spots, and show reduced sugar content, poor flavor, and shortened shelf life, rendering them unmarketable. The disease is highly dynamic epidemiologically, spreading rapidly through grafting, infected propagation material, and non-persistent aphid transmission. Foliar symptoms typically appear first and include chlorotic spots, vein clearing, mottling, banding, and leaf deformation, whereas fruit symptoms manifest as ring-shaped discolorations, necrotic areas, and structural abnormalities [5].

The physiological impact of PPV extends beyond visible symptoms to fundamental metabolic processes in the host. Numerous studies have shown that viral infections, including PPV, markedly alter photosynthetic efficiency, particularly at the level of photosystem II (PSII). Reduced electron transport rates, impaired photochemical efficiency (F_v_/F_m_), and decreased chlorophyll content are commonly observed in infected tissues, reflecting disruption of chloroplast structure and function that is often linked to oxidative damage and altered redox homeostasis [6,7].

A key mechanism underlying virus-induced physiological disruption is the overproduction of ROS. PPV infection triggers oxidative bursts in host cells, elevating hydrogen peroxide (H_2_O_2_) and other ROS. To counteract oxidative stress, plants have evolved sophisticated antioxidant systems comprising enzymatic and non-enzymatic components. The enzymatic machinery comprises several sequentially acting components: superoxide dismutase (SOD) converts superoxide radicals into H_2_O_2_, which catalase (CAT) and ascorbate peroxidase (APX) subsequently reduce to water and oxygen. APX is particularly important in chloroplasts, where it uses AsA as an electron donor in the AsA–glutathione (GSH) cycle [8]. The present study focused on this H_2_O_2_-detoxifying step and therefore quantified CAT and APX activities together with the ascorbate–glutathione pool; superoxide dismutase activity was not determined.

Despite extensive knowledge of PPV pathology and plant oxidative stress, the effects of PPV infection on the subcellular antioxidant network of woody perennial hosts under natural field conditions remain unclear. In particular, the coordinated responses of enzymatic and non-enzymatic antioxidants, photosynthetic pigments, and oxidative stress biomarkers in field-infected *Prunus* species have received little attention. Furthermore, most previous studies have relied on herbaceous model plants or controlled experimental systems, leaving integrated physiological and biochemical analyses under field conditions largely unexplored [5].

Recent advances in molecular diagnostics, including ELISA, immunochromatographic assays, and high-throughput sequencing (HTS), have substantially improved virus detection and characterization in *Prunus*. HTS approaches such as RNA sequencing (RNA-seq) allow simultaneous detection of multiple viral agents and the discovery of novel viruses. Surveys of peach orchards in different regions have revealed complex viral communities, including *Peach latent mosaic viroid*, *Prunus necrotic ringspot virus*, and other newly identified agents, underscoring the complexity of viral infections in stone fruit ecosystems [9]. Nevertheless, serological methods such as DAS-ELISA remain widely used for routine diagnosis because of their cost-effectiveness and reliability under field conditions [10].

In Azerbaijan, stone fruit production is central to horticultural systems, particularly in regions such as Quba where the climate favors *Prunus* cultivation [11]. Although PPV-induced oxidative stress has been documented in herbaceous models and several fruit crops, comprehensive field-based evaluations that integrate molecular detection with physiological and biochemical responses in naturally infected woody *Prunus* species remain limited [12,13]. To our knowledge, the present study is among the first to combine serological and molecular confirmation of PPV with an integrated assessment of oxidative stress, antioxidant defense, and photosynthetic performance in naturally infected plum, apricot, and peach trees under field conditions in Azerbaijan, providing region-specific baseline data on host responses across three commercially important *Prunus* species.

Accordingly, this study aimed to (i) determine the occurrence and distribution of PPV in major stone fruit-growing regions of Azerbaijan, (ii) confirm infection using serological and molecular methods, and (iii) characterize PPV-induced physiological and biochemical changes, focusing on oxidative stress markers, antioxidant enzyme activities, photosynthetic pigment composition, and redox homeostasis in infected *Prunus* species. This integrated approach provides new insights into host–virus interactions and improves understanding of oxidative stress mechanisms in perennial woody plants under natural infection.

## 2. Results and Discussion

### 2.1. Field Survey and Symptom Development

**Figure 1:**
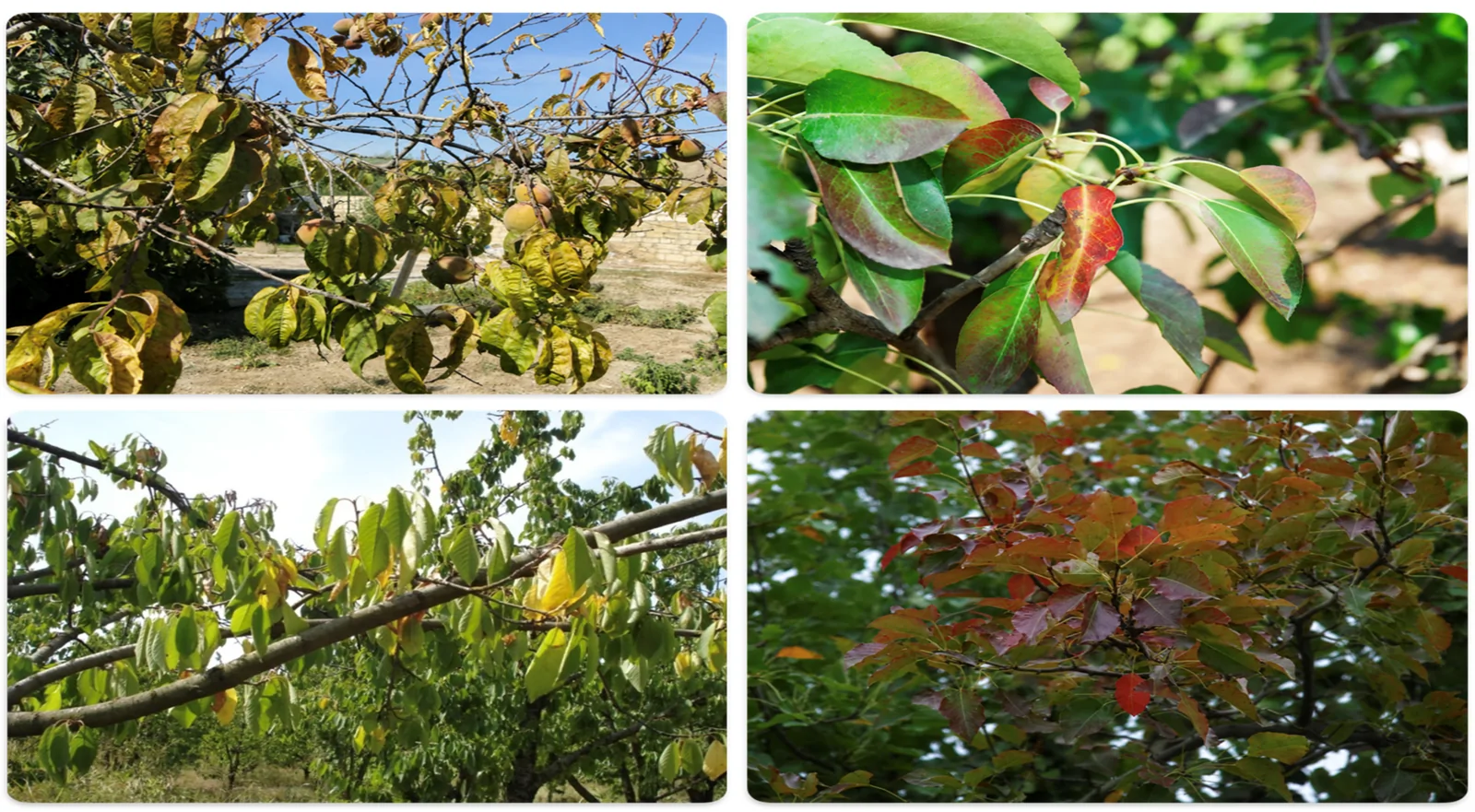
Foliar symptoms associated with PPV infection observed in stone fruit trees under field conditions in Azerbaijan. Infected *Prunus* spp. trees exhibit characteristic disease symptoms, including leaf curling, chlorotic and necrotic discoloration, mosaic patterns, vein deformation, and premature leaf senescence. Symptom severity varies among cultivars and is influenced by environmental conditions and disease progression stage.

Systematic field surveys were conducted during the 2024 growing season across the main production areas of the Quba district, Azerbaijan, to monitor the occurrence and distribution of viral infections in cultivated *Prunus* species. Orchards containing plum (*P. domestica* L.) cultivars “Imperial” and “Adi Vengerka”, peach (*P. persica* (L.) Batsch) cultivars “Fadai” and “Venus”, and apricot (*P. armeniaca* L.) cultivars “Parnas”, “Badami”, and “Shalakh” were included in the survey. These commercially important and susceptible cultivars were selected to ensure representative sampling across the region. Symptom expression was monitored from April to August to capture disease progression under field conditions. At the onset of flowering in early April, preliminary visual assessment revealed PPV-associated symptoms on floral tissues: infected blossoms showed diffuse chlorotic spotting and mosaic-like discoloration of petals of variable intensity, indicating early systemic viral activity in reproductive tissues. The first clearly distinguishable foliar symptoms appeared during the vegetative–reproductive transition in June, when infected leaves displayed characteristic Sharka symptoms, including chlorotic rings, vein clearing, mottling, and mild deformation (Figure 1).

These symptoms intensified as the season progressed, reflecting increased viral replication and systemic spread. During fruit development and ripening, symptom expression became more pronounced and cultivar-dependent. In mid-summer (July–August), infected fruits showed typical PPV-induced abnormalities, including ring-shaped chlorotic lesions, uneven coloration, and, in some cases, tissue deformation. Symptom severity was greater in late-ripening cultivars, consistent with prolonged virus–host interaction and cumulative physiological stress. Notably, blossom symptoms were the earliest and most reliable visual indicator of PPV infection under field conditions and were readily detected during routine orchard inspection at flowering, suggesting that floral tissues can serve as a practical target for early screening. The intensity of blossom symptoms varied among trees, reflecting differences in cultivar susceptibility, environmental conditions, and potential viral strain variability. The seasonal account above describes the visual symptom survey. All physiological and biochemical analyses reported in Section 2.3, Section 2.4, Section 2.5, Section 2.6 and Section 2.7 were performed on leaves collected at a single standardised sampling at the vegetative growth stage (June 2024), so that infected and healthy trees were compared at an identical phenological stage.

**Figure 2:**
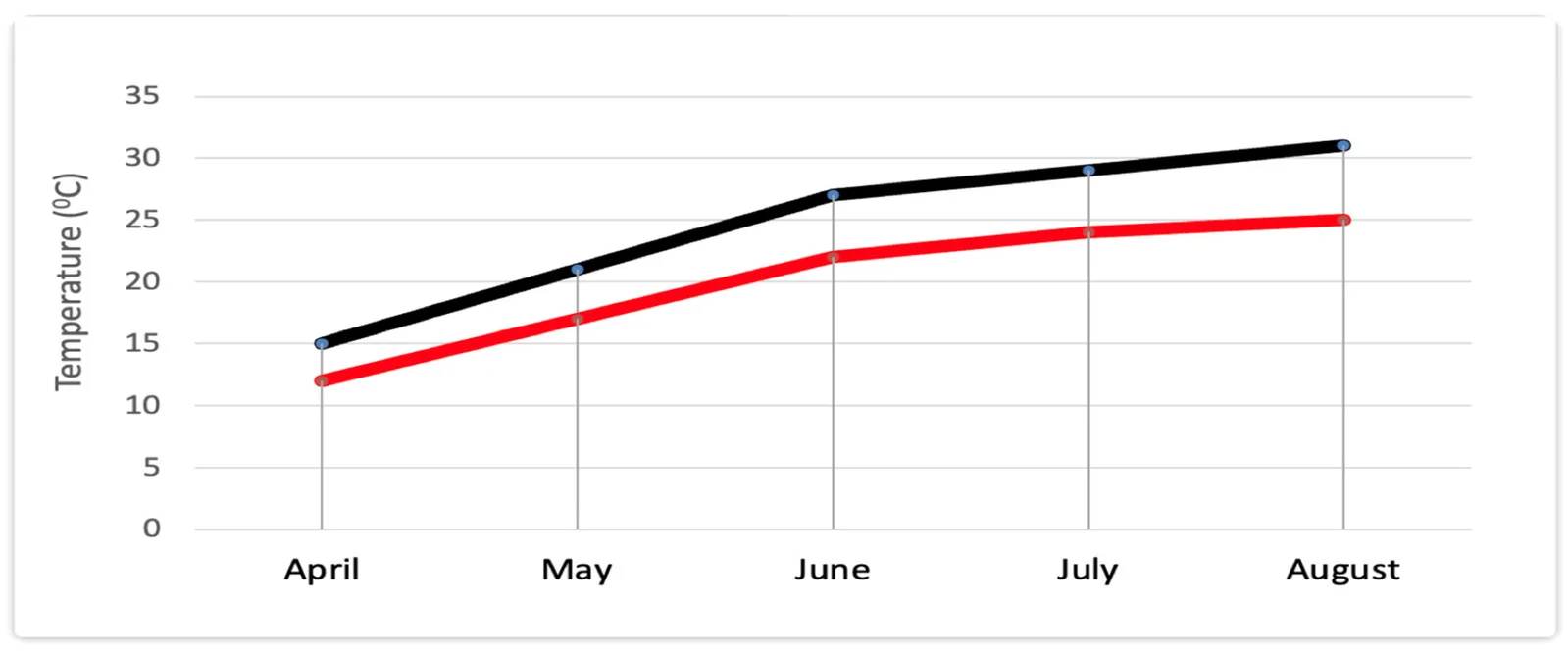
Monthly minimum and maximum air temperatures in Quba (Azerbaijan) during the growing season (April–August 2024). The plot shows monthly maximum (black line) and minimum (red line) temperatures (°C) for April through August, representing typical climatic conditions in the study region.

Climatic conditions during sampling followed a typical temperate–continental pattern with pronounced seasonal variation. In early spring (April), mean maximum and minimum air temperatures were approximately 15 °C and 12 °C, respectively, favoring the onset of vegetative growth and early symptom expression. Progressive warming continued through the season, and the highest temperatures were recorded in August, when mean maximum and minimum values reached approximately 31 °C and 25 °C (Figure 2).

**Figure 3:**
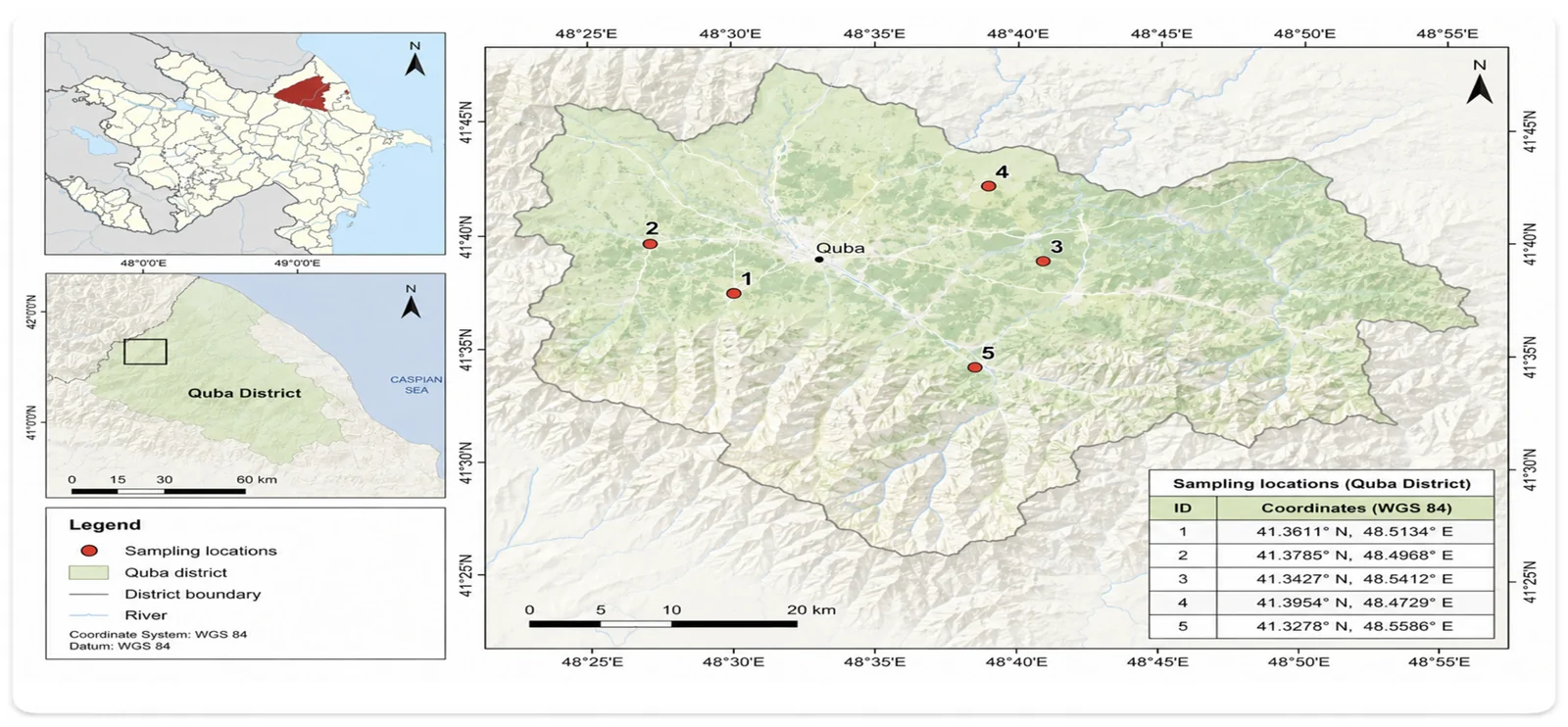
Map of the study area showing the locations of stone fruit orchards surveyed for PPV infection in Azerbaijan. Sampling sites (1–5) represent commercial orchards cultivated with peach (*P. persica*), apricot (*P. armeniaca*), and plum (*P. domestica*) in the Quba region. In the inset map of Azerbaijan the area shaded in red indicates the Quba district, in which all surveyed orchards were located; the red circles on the main map mark the five sampling sites (1–5).

The sampling orchards lie within the main fruit-growing zone of the Quba district, one of the most important horticultural regions of Azerbaijan, characterized by intensive cultivation of peach, plum, apricot, apple, and other temperate fruit crops (Figure 3).

### 2.2. Serological and Molecular Detection of PPV

Although RT-PCR is among the most sensitive methods for PPV detection, ELISA remains widely used and cost-effective for large-scale screening of stone fruit trees in epidemiological surveys [14]. PPV infection was initially screened using a double-antibody sandwich enzyme-linked immunosorbent assay (DAS-ELISA) and an AgriStrip one-step immunochromatographic assay. Of the 60 *Prunus* leaf samples analyzed, 22 tested positive for PPV, corresponding to a PPV detection rate of 36.7% among the symptomatic trees sampled. PPV-positive samples were detected in three host species, including *P. domestica* (plum) cultivar “Adi Vengerka”, *P. persica* (peach) cultivar “Fadai”, and *P. armeniaca* (apricot) cultivar “Shalakh”, demonstrating the occurrence of PPV in commercially cultivated stone fruit trees in the studied agroecosystems of the Quba region. Because sampling was directed at symptomatic trees and the numbers examined per species and cultivar were not balanced, these values describe the composition of the present sample and should not be interpreted as estimates of the relative susceptibility of the species or cultivars, which would require balanced randomised sampling and controlled inoculation.

**Figure 4:**
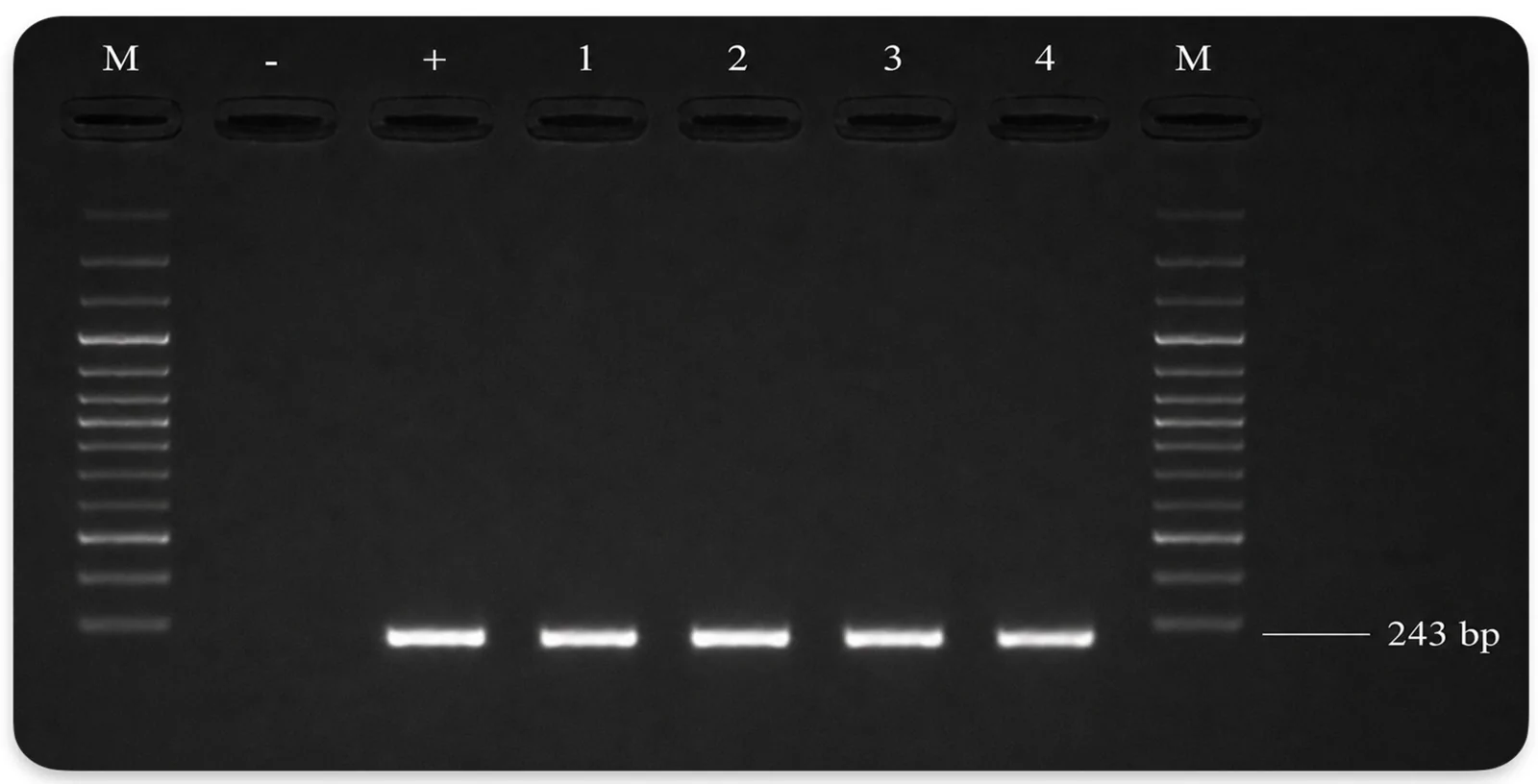
RT-PCR detection of PPV using the P1/P2 primer pair targeting the CP gene. Lane M: 2-Log DNA Ladder (New England Biolabs, Hitchin, UK); lane −: negative control (nuclease-free water); lane +: PPV-positive control; lanes 1–4: PPV-positive samples. An amplicon of the size expected for this primer pair (243 bp) was obtained from the positive control and from all infected samples, co-migrating with the amplicon of the PPV-positive reference isolate. PCR products were separated on a 1.5% agarose gel and visualized under UV illumination following ethidium bromide staining.

RT-PCR was performed using total RNA extracted from PPV-infected leaf tissues with primers specific to the PPV coat protein (CP) gene. Agarose gel electrophoresis revealed amplification of the expected 243-bp product (Figure 4), confirming the presence of PPV in the infected samples. The molecular-weight marker included in this experiment does not provide resolution in the size range of the amplicon; product identity was therefore established by the specificity of the PPV-specific primer pair and by co-migration with the amplicon obtained from the PPV-positive reference isolate, rather than by precise size estimation against the marker. A limitation of this study is that molecular characterization was restricted to detection by RT-PCR and did not extend to nucleotide sequencing of representative amplicons or strain identification; consequently, the PPV strains (e.g., PPV-D, PPV-Rec, PPV-M, or other variants) circulating in the surveyed orchards could not be determined. The primary objective was to confirm PPV infection and evaluate the associated physiological, biochemical, and oxidative stress responses rather than to characterize viral genetic diversity. Nevertheless, sequencing of representative amplicons followed by phylogenetic analysis and strain characterization would provide valuable epidemiological information on PPV populations in Azerbaijan and should be pursued in future work.

For subsequent biochemical and physiological analyses, representative PPV-infected trees with the highest DAS-ELISA absorbance values and clearly developed Sharka symptoms (leaf chlorosis, deformation, and mosaic patterns) were selected. Symptomless, DAS-ELISA-negative trees of the same species, of similar physiological age and grown under comparable conditions, served as healthy controls to enable direct comparison. Plant material was not stratified by cultivar; instead, infected trees were selected across the three *Prunus* species using uniform criteria of strong serological positivity and consistent symptom expression, thereby minimizing genotype-related selection bias. Each tree represented one biological replicate (three pooled leaves per tree), with five independent replicates per group (infected and healthy; n = 5), and each assay was performed in triplicate as technical replicates.

### 2.3. Oxidative Stress: H_2_O_2_ and Lipid Peroxidation

**Figure 5:**
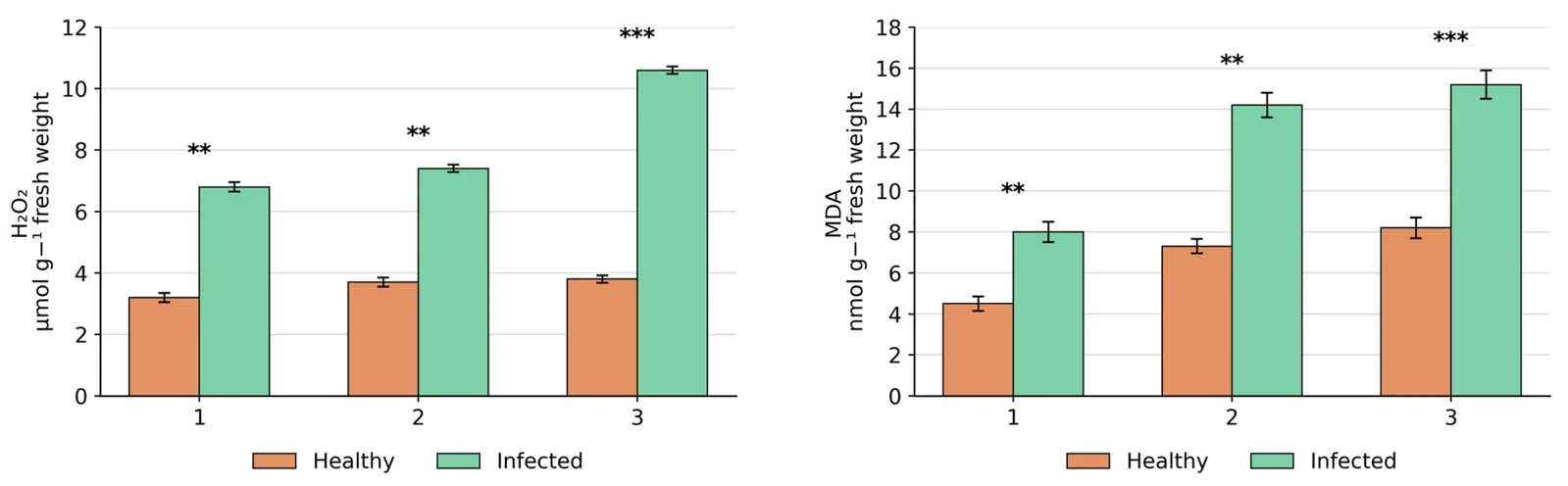
Changes in H_2_O_2_ concentration and lipid peroxidation in PPV-infected *Prunus* spp. leaves. H_2_O_2_ content (µmol g^−1^ FW; (**left**)) and MDA content (nmol g^−1^ FW; (**right**)) were determined in healthy and PPV-infected leaves of three *Prunus* species during the vegetative growth stage: (1) plum (*P. domestica*), (2) apricot (*P. armeniaca*), and (3) peach (*P. persica*). Data are presented as mean ± standard deviation (SD) of five independent biological replicates (*n* = 5). Statistical significance was determined using two-way analysis of variance (ANOVA) followed by Tukey’s honestly significant difference (HSD) post hoc test. Significance levels are shown directly above the bars.

PPV infection was associated with pronounced oxidative stress in all examined *Prunus* species, evidenced by significant increases in both H_2_O_2_ accumulation and lipid peroxidation relative to healthy controls (Figure 5). H_2_O_2_ concentration rose markedly in infected leaves of plum, apricot, and peach. H_2_O_2_ content was consistently higher in PPV-infected trees than in the corresponding healthy trees of each species, reaching approximately 6.8 µmol g^−1^ FW in plum, 7.4 µmol g^−1^ FW in apricot, and 10.6 µmol g^−1^ FW in peach, compared with 3.2–3.9 µmol g^−1^ FW in the healthy controls. The highest accumulation occurred in peach, nearly a 2.7-fold increase over the healthy control, indicating a stronger oxidative burst in this host. Differences between healthy and infected plants were significant in all species, most markedly in peach (*p* < 0.001).

A similar pattern was observed for MDA, a well-established indicator of lipid peroxidation and oxidative membrane damage. Healthy plants showed low MDA (≈4.5–8.2 nmol g^−1^ FW), whereas infected plants exhibited significantly elevated levels of approximately 8.0, 14.2, and 15.2 nmol g^−1^ FW in plum, apricot, and peach, respectively. The highest absolute MDA content was recorded in peach, almost twice that of the corresponding healthy control, whereas the largest increase relative to its own control was observed in apricot; both indicate marked membrane deterioration in infected trees.

Among species, peach showed the strongest biochemical response, followed by apricot and plum. The parallel rise in H_2_O_2_ and MDA suggests that excessive ROS generation promoted lipid peroxidation and membrane damage, disrupting cellular redox homeostasis in susceptible hosts. The positive association between H_2_O_2_ accumulation and MDA formation further supports a role for ROS-mediated membrane injury during PPV pathogenesis. These results agree with previous reports that PPV infection induces oxidative stress through ROS accumulation, increased H_2_O_2_ production, and enhanced lipid peroxidation in susceptible *Prunus* species; PPV-infected peach and apricot have been shown to exhibit elevated H_2_O_2_ together with increased oxidative damage and disruption of antioxidant defenses, ultimately leading to membrane deterioration and symptom development [15]. Moreover, Hernández et al. (2016) [16] emphasized that excessive ROS accumulation during compatible plant–virus interactions not only causes oxidative damage but also functions as a signaling mechanism that amplifies defense responses and regulates programmed cell death, thereby contributing to symptom severity and disease progression. The more pronounced oxidative damage observed in peach in the present study therefore suggests a lower capacity to maintain ROS homeostasis compared with apricot and plum, which may partly explain the greater susceptibility of peach to PPV infection [16].

### 2.4. Non-Enzymatic Antioxidants: GSH and AsA

**Figure 6:**
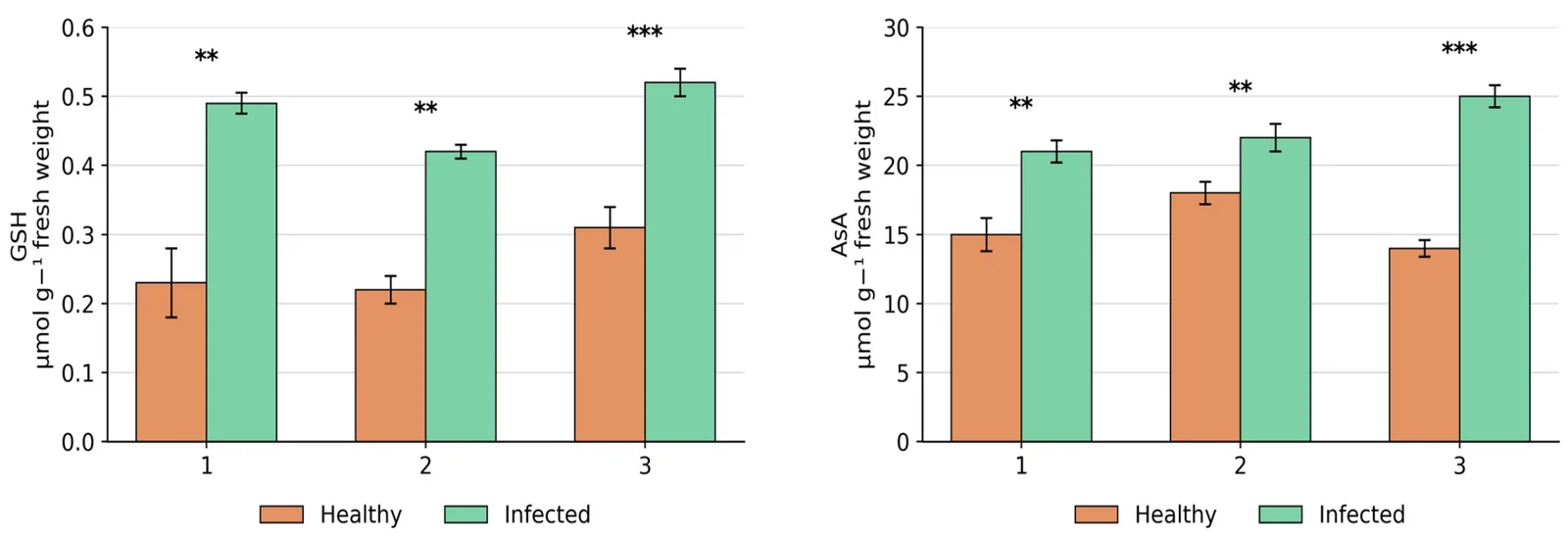
Changes in GSH and AsA concentrations in PPV-infected *Prunus* spp. leaves. GSH content (µmol g^−1^ FW; (**left**)) and AsA content (µmol g^−1^ FW; (**right**)) were determined in healthy and PPV-infected leaves of three *Prunus* species during the vegetative growth stage: (1) plum (*P. domestica*), (2) apricot (*P. armeniaca*), and (3) peach (*P. persica*). Data are presented as mean ± standard deviation (SD) of five independent biological replicates (*n* = 5). Statistical significance was assessed using two-way analysis of variance (ANOVA) followed by Tukey’s honestly significant difference (HSD) post hoc test. Significance levels are shown directly above the bars.

Reduced GSH and AsA were determined in healthy and infected leaves of plum, apricot, and peach during the vegetative growth stage (Figure 6). GSH content was significantly higher in infected trees of all species. In *P. domestica*, GSH was approximately 0.49 versus 0.23 µmol g^−1^ FW in healthy trees (*p* < 0.01; +113%); in *P. armeniaca*, 0.42 versus 0.22 µmol g^−1^ FW (*p* < 0.01; +91%); and the highest level was recorded in *P. persica* (0.52 vs. 0.31 µmol g^−1^ FW; *p* < 0.001; +68%). A comparable pattern was observed for AsA: infected *P. domestica* showed higher AsA (21 vs. 15 µmol g^−1^ FW; *p* < 0.01), *P. armeniaca* showed 22 versus 18 µmol g^−1^ FW (*p* < 0.01), and the most pronounced response occurred in *P. persica* (≈25 vs. 14 µmol g^−1^ FW; *p* < 0.001; +79%). Overall, both GSH and AsA were significantly higher in infected trees of all three species, with *P. persica* showing the strongest non-enzymatic antioxidant response.

The increases in GSH and AsA indicate activation of antioxidant defense pathways in response to pathogen-induced oxidative stress. Infection is commonly associated with excessive production of ROS, including H_2_O_2_ and superoxide radicals, which damage cellular structures unless neutralized [17]; plants respond by enhancing the synthesis and regeneration of low-molecular-weight antioxidants such as GSH and AsA to preserve redox homeostasis. GSH is central to the cellular antioxidant network, functioning in ROS scavenging, detoxification, and stress signaling [18]; the substantial rise in GSH in infected plants suggests an enhanced capacity to mitigate oxidative damage and maintain cellular integrity. Similar increases have been reported under biotic and abiotic stresses, where GSH improves tolerance by regulating redox status and activating defense pathways [17,19,20]. Likewise, AsA accumulation reflects its role as a primary non-enzymatic antioxidant that directly scavenges ROS and serves as an essential substrate in the AsA–GSH cycle. Elevated AsA has been linked to enhanced resistance to oxidative damage caused by pathogens, heavy metals, and environmental stresses [21,22]. Collectively, activation of the AsA–GSH cycle appears to be an important adaptive strategy for maintaining redox balance and improving tolerance under pathogen stress.

### 2.5. Enzymatic Antioxidants: CAT and APX

**Figure 7:**
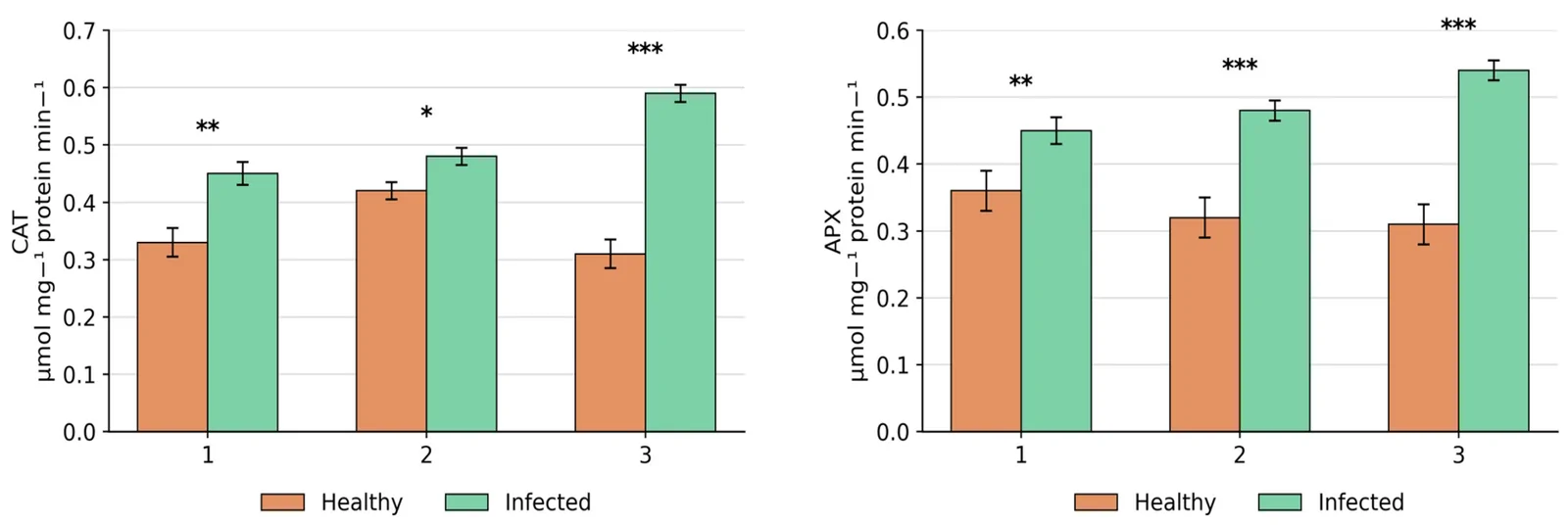
Activities of CAT (µmol mg^−1^ protein min^−1^; (**left**)) and APX (µmol mg^−1^ protein min^−1^; (**right**)) in healthy control and PPV-infected *Prunus* spp. leaves during the vegetative growth stage: (1) plum (*P. domestica*), (2) apricot (*P. armeniaca*), and (3) peach (*P. persica*). Data are presented as mean ± standard deviation (SD) of five independent biological replicates (*n* = 5). Statistical significance was assessed using two-way analysis of variance (ANOVA) followed by Tukey’s honestly significant difference (HSD) post hoc test. Significance levels are shown directly above the bars.

CAT and APX activities were evaluated in healthy and infected leaves of the three *Prunus* species during the vegetative growth stage (Figure 7). Both enzyme activities were significantly higher in infected trees, although the magnitude varied among species. CAT activity was significantly higher in infected plants of all species: in *P. domestica* it was approximately 0.45 versus 0.33 µmol mg^−1^ protein min^−1^ in healthy trees (*p* < 0.01; +32%); in *P. armeniaca*, 0.48 versus 0.42 µmol mg^−1^ protein min^−1^ (*p* < 0.05; +14%); and the highest activity was recorded in *P. persica* (0.59 vs. 0.31 µmol mg^−1^ protein min^−1^; *p* < 0.001; +90%). APX followed a similar trend: activity was approximately 0.45 versus 0.36 µmol mg^−1^ protein min^−1^ in *P. domestica* (*p* < 0.01; +25%), 0.48 versus 0.32 µmol mg^−1^ protein min^−1^ in *P. armeniaca* (*p* < 0.001; +50%), and 0.54 versus 0.31 µmol mg^−1^ protein min^−1^ in *P. persica* (*p* < 0.001; +74%).

Among the studied species, *P. persica* exhibited the strongest enzymatic antioxidant response, with the highest CAT and APX activities, whereas *P. armeniaca* showed the weakest CAT induction despite a substantial increase in APX activity, suggesting species-specific differences in antioxidant regulation. Overall, antioxidant enzyme activities were significantly higher in infected trees of all three *Prunus* species during the vegetative growth stage, reflecting activation of the enzymatic antioxidant defense system in response to virus-induced ROS accumulation. Catalase and ascorbate peroxidase act cooperatively to detoxify H_2_O_2_, thereby limiting oxidative damage and maintaining cellular redox balance. However, when considered together with the simultaneous increase in MDA, the present findings indicate that although antioxidant enzyme activities were markedly enhanced, they were insufficient to completely counteract excessive ROS production and lipid peroxidation. This suggests that oxidative stress exceeded the detoxification capacity of the antioxidant system, particularly in the more susceptible host species. Similar observations have been reported in PPV-infected *Prunus* species and other compatible plant–virus interactions, where activation of CAT and APX represents an adaptive defense response but does not fully prevent virus-induced oxidative damage [23]. A further limitation of the present study is that the upstream step of the enzymatic cascade, catalysed by superoxide dismutase, was not measured. Because SOD governs the rate at which superoxide is converted to H_2_O_2_, its determination, together with glutathione reductase, dehydroascorbate reductase and monodehydroascorbate reductase of the ascorbate–glutathione cycle, would provide a more complete description of the antioxidant network in PPV-infected stone fruit trees, and is planned for future work.

### 2.6. Photosynthetic Pigments and Protein Content

The effects of PPV infection on total chlorophyll, carotenoid content, Chlorophyll Stability Index (CSI), and protein content are summarized in Table 1. Total chlorophyll was lower in infected trees of all species relative to healthy controls: content ranged from 4.25 to 4.32 mg g^−1^ FW in healthy plants and from 2.94 to 3.54 mg g^−1^ FW in infected plants. The greatest reduction occurred in *P. armeniaca* (from 4.25 to 2.94 mg g^−1^ FW; 30.8%), followed by *P. domestica* (from 4.32 to 3.31 mg g^−1^ FW; 23.4%), while the smallest reduction was observed in *P. persica* (from 4.28 to 3.54 mg g^−1^ FW; 17.3%).

Carotenoid content was less affected than chlorophyll. A slight decrease was observed in *P. domestica* (1.68 to 1.58 mg g^−1^ FW) and *P. armeniaca* (1.62 to 1.54 mg g^−1^ FW), whereas *P. persica* showed a slight increase (1.69 to 1.72 mg g^−1^ FW). The maintenance and slight rise in carotenoids in peach, the species with the highest chlorophyll stability, is consistent with a protective role for these pigments in quenching reactive oxygen species and stabilizing the photosynthetic apparatus under viral stress, and may contribute to its comparatively lower pigment loss. The CSI ranged from 68.06% to 81.94%. The lowest value was recorded in *P. armeniaca* (68.06%), indicating the greatest chlorophyll degradation, whereas *P. persica* showed the highest CSI (81.94%), followed by *P. domestica* (76.62%), reflecting greater pigment preservation. Protein content was also lower in infected trees: healthy plants contained 9.38–9.58 mg g^−1^ FW, whereas infected plants ranged from 7.88 to 8.45 mg g^−1^ FW. The greatest reduction occurred in *P. domestica* (9.38 to 7.88 mg g^−1^ FW; 16.0%), followed by *P. armeniaca* (9.42 to 8.24 mg g^−1^ FW; 12.5%) and *P. persica* (9.58 to 8.45 mg g^−1^ FW; 11.8%).

Overall, photosynthetic pigments and soluble protein were lower in infected trees of all species. Among them, *P. armeniaca* exhibited the strongest reduction in chlorophyll content and the lowest chlorophyll stability, whereas *P. persica* maintained relatively higher pigment stability under infection. Similar findings have been reported, with virus infection causing significant alterations in photosynthetic performance, pigment degradation, and disturbances in primary metabolism [24,25]. Reductions in chlorophyll and protein have been linked to virus-induced oxidative stress and impairment of the photosynthetic apparatus, resulting in decreased physiological activity and vigor. These observations indicate that the severity of PPV-induced physiological damage varies among host species and genotypes, reflecting differences in tolerance and defense capacity.

### 2.7. Chlorophyll Fluorescence and PSII Efficiency

Chlorophyll fluorescence analysis showed that PPV infection was associated with reduced PSII photochemical efficiency in all species (Table 2). Compared with healthy plants, infected leaves showed reduced maximum quantum yield of PSII photochemistry (F_v_/F_m_), indicating impairment of the photosynthetic apparatus under viral stress. The decline in F_v_/F_m_ was accompanied by an increase in F_0_ and a decrease in F_m_, which together lowered variable fluorescence (F_v_ = F_m_ − F_0_) and reflected reduced efficiency of excitation energy conversion within PSII reaction centers, changes generally associated with disturbances in the electron transport chain. The magnitude of the F_v_/F_m_ reduction differed among species, indicating species-specific responses, with the lowest values in the most severely affected plants, consistent with greater photoinhibition.

Species that maintained relatively higher F_v_/F_m_ preserved PSII function more effectively under viral stress. Overall, the fluorescence data indicate that PPV infection is associated with impairment of primary photosynthetic processes in *Prunus* species. The reduction in PSII photochemical efficiency parallels the decreases in chlorophyll content and CSI in infected leaves, indicating that infection disrupts both pigment integrity and photosynthetic electron transport during the vegetative growth stage.

The decline in F_v_/F_m_ indicates reduced maximum quantum efficiency of PSII photochemistry and substantial stress on the photosynthetic apparatus. A decrease in F_v_/F_m_ is a sensitive indicator of photoinhibition and PSII dysfunction under biotic stress. The concurrent increase in F_0_ and decrease in Fm imply damage to PSII reaction centers and disruption of energy transfer within thylakoid membranes, potentially resulting from virus-induced oxidative stress that alters membrane integrity and the organization of photosynthetic complexes. The accompanying reductions in chlorophyll content and stability further support the hypothesis that infection compromises chloroplast structure and function [25,26]. At the same time, the enhanced CAT and APX activities and increased AsA and GSH contents in infected leaves indicate activation of antioxidant defenses to counteract excess ROS. Similar responses have been documented in numerous plant–virus and plant–pathogen interactions, where upregulation of antioxidant enzymes and accumulation of non-enzymatic antioxidants protect against oxidative damage and help maintain redox homeostasis and stress tolerance [27,28,29,30]. Despite this response, the decline in F_v_/F_m_ indicates that oxidative damage was sufficient to impair PSII photochemistry, particularly in the more susceptible species.

### 2.8. Correlation Analysis

**Figure 8:**
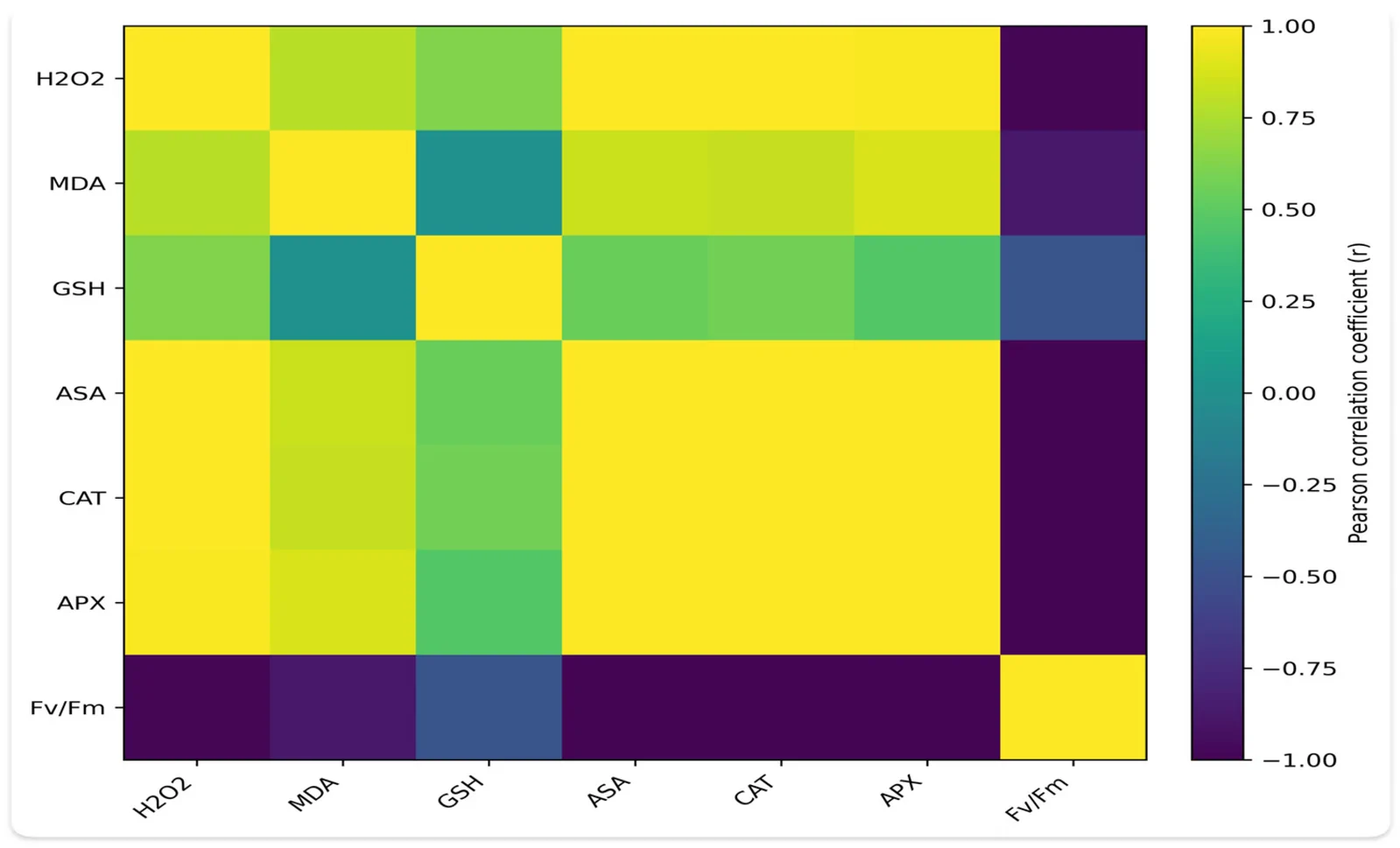
Pearson correlation heatmap illustrating relationships among oxidative stress markers (H_2_O_2_ and MDA), antioxidant defense components (GSH, AsA, CAT, and APX), and photosynthetic performance (F_v_/F_m_) in healthy and PPV-infected *Prunus* species. Pearson correlation coefficients (r) were calculated from five independent biological replicates per experimental group. Color intensity represents correlation strength, with yellow indicating strong positive correlations (r approaching +1), green indicating weak or moderate correlations, and purple indicating strong negative correlations (r approaching −1). Abbreviations: H_2_O_2_, hydrogen peroxide; MDA, malondialdehyde; GSH, reduced glutathione; AsA, ascorbic acid; CAT, catalase; APX, ascorbate peroxidase; F_v_/F_m_, maximum quantum efficiency of photosystem II.

Pearson’s correlation analysis was performed to investigate the relationships among oxidative stress markers, antioxidant defense components, and photosynthetic parameters in PPV-infected *Prunus* species (Figure 8).

The heatmap revealed predominantly positive associations among the oxidative stress and antioxidant parameters (H_2_O_2_, MDA, AsA, GSH, CAT, and APX), indicating coordinated activation of enzymatic and non-enzymatic defenses in response to infection. Increased H_2_O_2_ and MDA were associated with enhanced antioxidant responses, suggesting that virus-induced oxidative stress triggered protective cellular mechanisms [31,32,33]. In contrast, F_v_/F_m_ showed an inverse relationship with most oxidative stress and antioxidant parameters, indicating that greater oxidative damage was accompanied by a decline in PSII performance, consistent with the reductions in chlorophyll content and fluorescence parameters detected in infected plants.

These correlations should be interpreted with caution given the relatively small number of biological replicates. Although they provide insight into the relationships among oxidative stress markers, antioxidant defense components, and photosynthetic performance, larger sample sizes are needed to confirm their robustness and statistical significance. Overall, the correlation analysis supports the conclusion that PPV infection is associated with oxidative stress and antioxidant defense responses and with reduced photosynthetic activity in *Prunus* species.

This study was conducted on naturally infected, mature trees under field conditions. Because no controlled inoculation was performed and viral load was not quantified, the physiological and biochemical differences reported here should be interpreted as associations with PPV infection status rather than as effects attributable exclusively to PPV. Although infected and healthy trees were paired within orchards and matched for species, age class and sampling conditions, a contribution of co-occurring biotic and abiotic stressors, including possible mixed infection with other *Prunus*-infecting viruses and viroids, which was not assessed in this work, cannot be excluded. Quantitative RT-PCR or droplet digital PCR to relate viral titre to the magnitude of the oxidative response, high-throughput sequencing to exclude mixed infections, and controlled graft inoculation of standardised rootstocks under insect-proof conditions are required to establish causality, and are planned as the next stage of this work. In addition, because the biochemical and photosynthetic measurements were obtained at a single phenological stage, the present data describe the physiological status of infected trees at the vegetative growth stage and do not resolve the time course of the oxidative response.

### 2.9. Sustainable Development Goals (SDG) Statement

This study contributes to the United Nations Sustainable Development Goals, particularly SDG 2 (Zero Hunger) and SDG 15 (Life on Land). Detection and molecular confirmation of PPV in economically important stone fruit species (*P. domestica*, *P. armeniaca*, and *P. persica*) provide essential information for improving plant disease monitoring and management. By reducing virus-induced yield losses and supporting early diagnosis, the findings promote sustainable fruit production and food security, in line with SDG 2. Assessment of PPV-induced oxidative stress, photosynthetic impairment, and metabolic alterations also advances understanding of plant–pathogen interactions and plant health under biotic stress, supporting the conservation of agricultural biodiversity and the sustainable management of land ecosystems in accordance with SDG 15.

## 3. Materials and Methods

### 3.1. Plant Material

Field surveys were conducted during the 2024 growing season in Quba, the main stone fruit-producing region of Azerbaijan. Orchards of plum (*P. domestica* L.), apricot (*P. armeniaca* L.), and peach (*P. persica* (L.) Batsch) were inspected for Sharka symptoms. Typical field symptoms included chlorotic rings, mottling, vein clearing, leaf deformation, diffuse yellowing, and chlorotic spots (Figure 1). Sixty leaf samples were collected from mature trees (8–12 years old) showing characteristic PPV symptoms. Sampling was performed between 09:00 and 11:00 under dry conditions to minimize diurnal variation in physiological and biochemical parameters. Meteorological data for the growing season indicated progressive seasonal warming in the study area (Figure 2). The surveyed orchards were located at 41.3611° N, 48.5134° E; 41.3785° N, 48.4968° E; 41.3427° N, 48.5412° E; 41.3954° N, 48.4729° E; and 41.3278° N, 48.5586° E (Figure 3).

From each tree, three fully expanded, physiologically mature leaves were collected from different canopy positions and pooled into a single biological sample. Thus, each tree constituted one biological sample and one independent biological replicate, whereas the individual leaves were treated as subsamples of the same biological unit rather than independent replicates. Symptomless leaves from healthy trees in the same orchards served as controls. Leaves for the physiological and biochemical analyses were collected in a single standardised sampling at the vegetative growth stage (June 2024). Samples were initially collected from five orchard sites. For the final analysis, five biological replicates per group (PPV-infected and healthy) with confirmed infection status and complete biochemical datasets were included (*n* = 5 per group). Samples lacking molecular confirmation of PPV infection or failing quality requirements for biochemical analysis were excluded. Accordingly, all biochemical and statistical analyses were performed using five independent biological replicates (individual trees) per group. Immediately after collection, samples were transported to the Laboratory of Bioadaptation, Institute of Molecular Biology and Biotechnologies, under refrigerated conditions. Samples for virus detection were stored at 4 °C and processed within 24 h, whereas samples designated for biochemical analyses were immediately frozen in liquid nitrogen and stored at −80 °C until analysis.

### 3.2. Serological Detection of PPV by ELISA

Leaf samples were screened for PPV using immunochromatographic assays and DAS-ELISA. Rapid screening used AgriStrip PPV test strips (BIOREBA AG, Reinach, Switzerland) according to the manufacturer’s instructions: approximately 100 mg of leaf tissue was homogenized in the supplied extraction buffer, test strips were immersed in the extract, and results were read after 10–15 min. The appearance of both control and test lines was scored as positive. Serological confirmation was performed with commercial DAS-ELISA kits (BIOREBA AG, Reinach, Switzerland) following Clark and Adams (1977) [21]. Leaf samples were homogenized in extraction buffer at 1:20 (*w*/*v*); microtiter plates coated with PPV-specific antibodies were incubated with the plant extracts and then with enzyme-conjugated antibodies. Absorbance was measured at 405 nm using a Stat Fax microplate reader (Awareness Technology Inc., Palm City, FL, USA). Samples were considered positive when absorbance exceeded at least twice that of healthy controls.

### 3.3. Molecular Detection of PPV by RT-PCR

PPV infection was confirmed using a sensitive, polyvalent reverse-transcription polymerase chain reaction (RT-PCR) assay, with positive and negative controls included in every run. Total RNA was extracted from 100 mg of leaf tissue using TRIzol reagent (Invitrogen, Thermo Fisher Scientific, Carlsbad, CA, USA). Leaf samples were ground into a fine powder in liquid nitrogen and processed according to the manufacturer’s instructions. The extracted RNA was stored at −80 °C, and its concentration and purity were determined using a NanoDrop spectrophotometer (Thermo Fisher Scientific, Wilmington, DE, USA). One-step RT-PCR was performed using the Verso 1-Step RT-PCR Kit (Thermo Fisher Scientific, Waltham, MA, USA) in a final reaction volume of 25 µL. The universal PPV-specific primers of Wetzel et al. (1991) [34], widely regarded as reference primers for broad-spectrum PPV detection because of their high sensitivity and specificity [10], were used: P2 (forward) 5′-CAG ACT ACA GCC TCG CCA GA-3′ and P1 (reverse) 5′-ACC GAG ACC ACT ACA CTC CC-3′. RT-PCR was performed under the following cycling conditions: reverse transcription at 50 °C for 30 min, initial denaturation at 94 °C for 3 min, followed by 45 cycles of denaturation at 94 °C for 1 min, annealing at 55 °C for 1 min, and extension at 72 °C for 1 min, with a final extension at 72 °C for 5 min. Amplified products were resolved by electrophoresis on 1.5% agarose gels prepared in 1× TBE buffer, stained with ethidium bromide, and visualized under UV illumination using a gel documentation system (Syngene, Cambridge, UK). Fragment sizes were estimated using a 2-Log DNA Ladder (New England Biolabs, Hitchin, UK). Amplification of the expected 243 bp product confirmed PPV infection. A PPV-positive reference isolate and nuclease-free water were used as positive and negative controls, respectively.

### 3.4. Quantification of H_2_O_2_

The H_2_O_2_ content was determined according to the method described by Velikova et al. (2000) [35]. Fresh leaf tissue (0.5 g) was homogenized in ice-cold 0.1% (*w*/*v*) trichloroacetic acid (TCA) and centrifuged at 12,000× *g* for 15 min at 4 °C. The supernatant was mixed with potassium phosphate buffer (pH 7.0) and potassium iodide, and the absorbance was measured at 390 nm. The H_2_O_2_ concentration was calculated using a standard calibration curve.

### 3.5. Lipid Peroxidation

Lipid peroxidation was quantified by measuring malondialdehyde (MDA) levels, an indicator of oxidative membrane damage, using a thiobarbituric acid (TBA) assay. Fresh leaf extracts were reacted with 0.5% TBA, and the absorbance was measured at 532 and 600 nm using a Evolution 350 UV/Vis spectrophotometer (Thermo Fisher Scientific, Madison, WI, USA). MDA content was calculated as A = (D_532_ − D_600_)/(E × m), where A is the MDA concentration, D is the absorbance, m is the fresh weight (FW), and E is the extinction coefficient [36].

### 3.6. Determination of Ascorbic Acid (AsA)

AsA content was determined according to the method described by Tayar et al. (2023) [37] based on its reducing capacity under acidic conditions. Fresh leaf tissue (0.5 g) was homogenized in 0.1 M sodium citrate buffer (pH 3.69) and centrifuged at 12,500× *g* for 5 min. The resulting supernatant was mixed with potassium ferricyanide (K_3_[Fe(CN)_6_]) and ferric chloride (FeCl_3_) to produce the characteristic Prussian blue complex. After incubation, absorbance was read at 697 nm (Evolution 350; Thermo Fisher Scientific, Madison, WI, USA), and AsA concentration was determined from a standard calibration curve.

### 3.7. Determination of Reduced Glutathione (GSH)

Reduced glutathione (GSH) content was determined spectrophotometrically at 412 nm using a commercial GSH assay kit (Sigma-Aldrich, St. Louis, MO, USA; Cat. No. CS0260). The reaction mixture consisted of 95 mM potassium phosphate buffer (pH 7.0), 0.038 mg mL^−1^ (48 µM) NADPH, 0.95 mM EDTA, 0.031 mg mL^−1^ 5,5′-dithiobis-(2-nitrobenzoic acid) (DTNB), 0.24% 5-sulfosalicylic acid, and 0.115 U mL^−1^ glutathione reductase. Glutathione standards (0.5–20 µg mL^−1^) were prepared in 0.4 M Tris-HCl buffer (pH 8.5) to generate a calibration curve.

### 3.8. Antioxidant Enzyme Assays

For enzyme extraction, 0.5 g of fresh leaf tissue was homogenized at 4 °C in extraction buffer containing 100 mM sodium phosphate (pH 7.8), 1 mM EDTA, 1% (*w*/*v*) PVP, 0.1% Triton X-100, and 2 mM PMSF. The homogenate was centrifuged at 12,000× *g* for 15 min at 4 °C, and the resulting supernatant was used for enzyme activity assays. Ascorbate peroxidase (APX) activity was determined by monitoring the decrease in absorbance at 290 nm resulting from AsA oxidation [38], using an extinction coefficient of 2.8 mM^−1^ cm^−1^. CAT activity was measured by monitoring the decomposition of H_2_O_2_ at 240 nm in a reaction mixture containing phosphate buffer, enzyme extract, and 3% H_2_O_2_ and was expressed as µmol H_2_O_2_ min^−1^ mg^−1^ protein using an extinction coefficient of 39.5 mM^−1^ cm^−1^ [39]. Soluble protein content was determined using the Bradford assay at 595 nm with bovine serum albumin (BSA) as the standard [40].

### 3.9. Photosynthetic Performance

The effects of PPV infection on the photosynthetic apparatus were evaluated by measuring chlorophyll fluorescence using a portable pulse-amplitude-modulated fluorometer (Mini-PAM; Walz, Effeltrich, Germany). The leaves were dark-adapted for 30 min to ensure complete oxidation of the photosystem II (PSII) reaction centers. Minimum fluorescence (F_0_) was recorded under weak modulated light, whereas maximum fluorescence (F_m_) was measured after a saturating light pulse. The maximum quantum efficiency of PSII photochemistry was calculated as F_v_/F_m_ = (F_m_ − F_0_)/F_m_, where F_v_ represents the variable fluorescence. A reduction in F_v_/F_m_ is an indicator of PPV-induced photoinhibition and impaired photosynthetic electron transport [41].

### 3.10. Photosynthetic Pigments

Photosynthetic pigment contents were determined spectrophotometrically according to the method described by McKinney (1941), with minor modifications [42]. Fresh leaf tissue (0.2 g) was homogenized in 80% (*v*/*v*) acetone and centrifuged at 10,000× *g* for 10 min. The absorbance of the resulting supernatant was measured at 662, 644, and 440.5 nm using an Ultrospec 3300 pro spectrophotometer (Amersham Biosciences, Arlington Heights, IL, USA). Chlorophyll a (Chl a), chlorophyll b (Chl b), total chlorophyll (Chl a + b), and carotenoid contents were calculated using the following equations:

Chl a = 9.784 × A662 − 0.990 × A644

Chl b = 21.426 × A644 − 4.650 × A662

Total chlorophyll = 5.134 × A662 + 20.436 × A644

Carotenoids = 4.695 × A440.5 − 0.268 × (Chl a + b)

Pigment concentrations were expressed as mg g^−1^ FW. Chlorophyll integrity was evaluated using the CSI [43]:

CSI (%) = (total chlorophyll in infected leaves/total chlorophyll in healthy leaves) × 100

Lower CSI values indicate greater chlorophyll degradation and damage to the photosynthetic apparatus.

### 3.11. Statistical Analyses

Statistical analyses were performed using R version 4.5.1 (R Foundation for Statistical Computing, Vienna, Austria). Data are presented as the mean ± standard deviation (SD) of five independent biological replicates (*n* = 5). The effects of *Prunus* species, PPV infection status, and their interaction on physiological and biochemical parameters were evaluated using two-way analysis of variance (ANOVA). When significant effects were detected, means were compared using Tukey’s honestly significant difference (HSD) post hoc test. Differences were considered statistically significant at *p* < 0.05. Pearson correlation analysis assessed relationships among oxidative stress markers, antioxidant defense components, and photosynthetic performance. The analyzed variables were H_2_O_2_, MDA, AsA, GSH, CAT, APX, and F_v_/F_m_. Correlation coefficients (r) and associated *p* values were computed with the *cor.test* function based on biological replicate data, and the resulting matrix was visualized as a heatmap.

## 4. Conclusions

This study confirmed the occurrence of PPV in stone fruit orchards of Azerbaijan and showed that infection is associated with substantial physiological and biochemical changes in susceptible *Prunus* species. Serological and molecular analyses verified PPV in peach (*P. persica*), apricot (*P. armeniaca*), and plum (*P. domestica*), with peach accounting for the largest number of positive samples in the present survey. Infection was associated with enhanced oxidative stress, increased H_2_O_2_ accumulation, and elevated MDA content, indicating intensified ROS production and membrane lipid peroxidation. In response, infected plants activated antioxidant defenses, increasing CAT and APX activities and accumulating AsA and GSH. Concurrent reductions in total chlorophyll, PSII photochemical efficiency (F_v/_F_m_), and soluble protein reflected the detrimental effects of PPV on photosynthesis and cellular metabolism.

Collectively, these findings indicate that PPV infection is associated with changes in both the biochemical and photochemical components of photosynthesis, and with reduced photosynthetic efficiency and altered redox homeostasis in stone fruit trees. Because the study was based on naturally infected trees, without controlled inoculation or quantification of viral load, these relationships should be regarded as associations rather than as effects attributable exclusively to PPV. The observed changes in antioxidant enzyme activity, oxidative stress biomarkers, and photosynthetic parameters may serve as physiological indicators for assessing host responses to PPV. As the causal agent of Sharka disease and one of the most economically damaging viral pathogens of stone fruit worldwide, PPV is regulated as a quarantine pest in many countries because of its efficient spread through infected propagation material and aphid vectors; its detection in Azerbaijani orchards therefore has important phytosanitary implications. Future work should prioritize nucleotide sequencing of representative amplicons and phylogenetic strain characterization to define the PPV variants circulating in Azerbaijani orchards, alongside continuous surveillance, early diagnostic approaches, regular monitoring, and the development of PPV-tolerant or resistant cultivars for sustainable stone fruit production and effective disease management.

## Figures and Tables

**Table 1:** Effects of PPV infection on photosynthetic pigments, protein content, and CSI in healthy and PPV-infected *Prunus* species. Values are presented as mean ± SD of five independent biological replicates (*n* = 5). Significant differences among the experimental groups were assessed using two-way analysis of variance (ANOVA), followed by Tukey’s honestly significant difference (HSD) post hoc test. Asterisks denote statistically significant differences between PPV-infected trees and the corresponding healthy controls (* *p* < 0.05).

Plant Sample	Total Chlorophyll (Chl a + Chl b) (mg g^−1^ FW)	Carotenoid Content (mg g^−1^ FW)	CSI (%)	Protein Content (mg g^−1^ FW)
Healthy control plum (*P. domestica*)	4.32 ± 0.30	1.68 ± 0.30	—	9.38 ± 0.30
PPV-infected plum (*P. domestica*)	3.31 ± 0.40 *	1.58 ± 0.20	76.62	7.88 ± 0.40 *
Healthy control apricot (*P. armeniaca*)	4.25 ± 0.30	1.62 ± 0.30	—	9.42 ± 0.30
PPV-infected apricot (*P. armeniaca*)	2.94 ± 0.20 *	1.54 ± 0.10 *	68.06	8.24 ± 0.50 *
Healthy control peach (*P. persica*)	4.28 ± 0.30	1.69 ± 0.30	—	9.58 ± 0.30
PPV-infected peach (*P. persica*)	3.54 ± 0.30 *	1.72 ± 0.10	81.94	8.45 ± 0.30 *

**Table 2:** Chlorophyll fluorescence parameters (F_0_, F_m_, and F_v_/F_m_) in healthy and PPV-infected stone fruit trees. Data are presented as mean ± standard deviation (SD) of five independent biological replicates (*n* = 5). Statistical significance was assessed using two-way analysis of variance (ANOVA) followed by Tukey’s honestly significant difference (HSD) post hoc test. Asterisks denote statistically significant differences between PPV-infected trees and the corresponding healthy controls (* *p* < 0.05).

Plant Sample	F_0_	F_m_	F_v_/F_m_
Healthy control plum (*P. domestica*)	0.252 ± 0.006	0.907 ± 0.018	0.722 ± 0.011
PPV-infected plum (*P. domestica*)	0.324 ± 0.008 *	0.738 ± 0.015 *	0.561 ± 0.012 *
Healthy control apricot (*P. armeniaca*)	0.245 ± 0.005	0.862 ± 0.017	0.716 ± 0.010
PPV-infected apricot (*P. armeniaca*)	0.348 ± 0.009 *	0.713 ± 0.014 *	0.512 ± 0.011 *
Healthy control peach (*P. persica*)	0.238 ± 0.006	0.876 ± 0.016	0.728 ± 0.012
PPV-infected peach (*P. persica*)	0.456 ± 0.011 *	0.770 ± 0.018 *	0.408 ± 0.009 *

## Data Availability

The original contributions presented in this study are included in the article. Further inquiries can be directed to the corresponding authors.

## Abbreviations

The following abbreviations are used in this manuscript:

ANOVA	Analysis of variance
APX	Ascorbate peroxidase
AsA	Ascorbic acid
BSA	Bovine serum albumin
CAT	Catalase
CP	Coat protein
CSI	Chlorophyll Stability Index
DAS-ELISA	Double-antibody sandwich enzyme-linked immunosorbent assay
FAO	Food and Agriculture Organization of the United Nations
FW	Fresh weight
GSH	Reduced glutathione
H_2_O_2_	Hydrogen peroxide
HSD	Honestly significant difference
HTS	High-throughput sequencing
MDA	Malondialdehyde
PPV	*Plum pox virus*
PSII	Photosystem II
ROS	Reactive oxygen species
RT-PCR	Reverse transcription polymerase chain reaction
SD	Standard deviation
SDG	Sustainable Development Goal
TCA	Trichloroacetic acid
